# Biological CO_2_ conversion to acetate in subsurface coal-sand formation using a high-pressure reactor system

**DOI:** 10.3389/fmicb.2013.00361

**Published:** 2013-12-02

**Authors:** Yoko Ohtomo, Akira Ijiri, Yojiro Ikegawa, Masazumi Tsutsumi, Hiroyuki Imachi, Go-Ichiro Uramoto, Tatsuhiko Hoshino, Yuki Morono, Sanae Sakai, Yumi Saito, Wataru Tanikawa, Takehiro Hirose, Fumio Inagaki

**Affiliations:** ^1^Kochi Institute for Core Sample Research, Japan Agency for Marine-Earth Science and Technology (JAMSTEC)Kochi, Japan; ^2^Submarine Resources Research Project, Japan Agency for Marine-Earth Science and Technology (JAMSTEC)Kochi, Japan; ^3^Civil Engineering Research Laboratory, Central Research Institute of Electric Power IndustryChiba, Japan; ^4^Institute of Biogeosciences, Japan Agency for Marine-Earth Science and Technology (JAMSTEC)Yokosuka, Japan

**Keywords:** geological CO_2_ sequestration, bituminous coal, geobio-reactor system, coal-bed methane, methanogen, homo-acetogenesis

## Abstract

Geological CO_2_ sequestration in unmineable subsurface oil/gas fields and coal formations has been proposed as a means of reducing anthropogenic greenhouse gasses in the atmosphere. However, the feasibility of injecting CO_2_ into subsurface depends upon a variety of geological and economic conditions, and the ecological consequences are largely unpredictable. In this study, we developed a new flow-through-type reactor system to examine potential geophysical, geochemical and microbiological impacts associated with CO_2_ injection by simulating *in-situ* pressure (0–100 MPa) and temperature (0–70°C) conditions. Using the reactor system, anaerobic artificial fluid and CO_2_ (flow rate: 0.002 and 0.00001 ml/min, respectively) were continuously supplemented into a column comprised of bituminous coal and sand under a pore pressure of 40 MPa (confined pressure: 41 MPa) at 40°C for 56 days. 16S rRNA gene analysis of the bacterial components showed distinct spatial separation of the predominant taxa in the coal and sand over the course of the experiment. Cultivation experiments using sub-sampled fluids revealed that some microbes survived, or were metabolically active, under CO_2-rich_ conditions. However, no methanogens were activated during the experiment, even though hydrogenotrophic and methylotrophic methanogens were obtained from conventional batch-type cultivation at 20°C. During the reactor experiment, the acetate and methanol concentration in the fluids increased while the δ^13^C_acetate_, H_2_ and CO_2_ concentrations decreased, indicating the occurrence of homo-acetogenesis. 16S rRNA genes of homo-acetogenic spore-forming bacteria related to the genus *Sporomusa* were consistently detected from the sandstone after the reactor experiment. Our results suggest that the injection of CO_2_ into a natural coal-sand formation preferentially stimulates homo-acetogenesis rather than methanogenesis, and that this process is accompanied by biogenic CO_2_ conversion to acetate.

## Introduction

In addition to causing dramatic increases in the surface temperature of the Earth, the release of anthropogenic greenhouse gasses into the atmosphere is considered to have had a major effect on changes to the oceans and climate (e.g., sea-level changes, ocean acidification) (Crowley, 2000; Alley et al., 2003; Karl and Trenberth, 2003). A variety of potential methods have been proposed to reduce anthropogenic CO_2_ emissions. Of the methods proposed to date, CO_2_ capture and storage followed by geologic CO_2_ sequestration (GCS) have been proposed as effective means to prevent the potentially dangerous consequences of climate change (White et al., 2003; Anderson and Newell, 2004; IPCC Special Reports, 2005; Davison, 2007; Zakkour and Haines, 2007; Benson and Cole, 2008; Kirk, 2011). As a result, a number of potential CO_2_ storage sites have been identified in a wide variety of geological settings, and feasibility assessments have been undertaken by some corporations and national governments (e.g., European Union, United States and Australia), which leads to draft a variety of legal instruments to accommodate commercial interest in GCS projects (White et al., 2004; Zakkour and Haines, 2007). One such example is the Utsira sandstone formation, an aquifer 800 m beneath the North Sea, into which 1 Mt of CO_2_ has been injected per year by Statoil since 1996 (Eiken et al., 2000).

Preventing CO_2_ leaking from geologic formations is an essential component of minimizing maintenance costs and avoiding the extensive environmental damage that would result from a large-scale leak. Some studies have examined admissible quantities of CO_2_ leakage and operational costs, as well as the efficiencies of impermeable cap-rock layers and sealing mechanisms (Anderson and Newell, 2004; Davison, 2007; Van der Zwaan and Gerlagh, 2009; Song and Zhang, 2013). To prevent CO_2_ leakage and reduce costs, the offshore subsurface has been considered as potential realm of GCS. In the deep subseafloor environment, CO_2_ can exist as a liquid/supercritical phase or it can dissolve into ambient seawater under conditions of high pressure and low temperature, facilitating migration into subsurface geologic formations. Since CO_2_ dissolved in seawater and supercritical CO_2_ are less dense than ambient seawater, they are buoyant after sequestration, whereas liquid CO_2_ has a higher density than ambient seawater, causing it to sink (House et al., 2006; Benson and Cole, 2008). However, at water depths of <3000 m and a few hundred meters of sediment, the generation of CO_2_ hydrate disturbs the seepage of high-density liquid CO_2_ through deep-sea sediments (House et al., 2006), suggesting superiority of deep-sea subsurface environment as a GCS site.

At present, the major techniques employed for trapping CO_2_ are capillary trapping, solubility trapping and mineral trapping (Mitchell et al., 2010; Jun et al., 2013). All of these mechanisms require empty space inside geological formations for storing gaseous/liquid CO_2_ or precipitated carbonates. Sandstones are considered to be well suited for GCS because of their high porosity and their ubiquity (Bachu, 2003). Hydrocarbon reservoirs (e.g., unmineable subsurface oil/gas fields and coal beds) have also been considered for use as potential geologic CO_2_ repositories. Recovery of coal-bed CH_4_ (CBM) associated with hydrocarbon reservoirs can be facilitated by CO_2_ injection, which potentially contributes to decreasing the energy costs associated with such a venture (Gunter et al., 1997; White et al., 2005).

The geophysical, geochemical and ecological impacts of CO_2_ sequestration in the natural subsurface environment have largely remained unknown. The injection of CO_2_ can promote the precipitation of carbonates through a reaction between the CO_2_ and surrounding rocks (Oelkers et al., 2008; Rosenbauer et al., 2012), probably decreasing rock porosity/permeability and resulting in secondary cap-rock generation (Kharaka et al., 2006), however, compared to capillary trapping or solubility trapping, carbonate precipitation occurs too slowly for it to be considered as an effective means of capturing CO_2_ (Gilfillan et al., 2009).

On the other hand, the changes in the chemical environment associated with GCS by CO_2_ injection can have a marked impact on the microbial consortia within sediments. Supercritical CO_2_ or water containing large amounts of dissolved CO_2_ kills microorganisms by disrupting their cell membranes (Bertoloni et al., 2006; Wu et al., 2010), whereas the maximum limits of microbial CO_2_ tolerance are still poorly understood. Numerous studies have confirmed that methanogens are capable of growth in aqueous media containing dissolved CO_2_ concentrations that are considerably higher than those of natural conditions (Yakimov et al., 2002; Videmsek et al., 2009; Oppermann et al., 2010). The microbiological and geochemical characteristics of the deep-sea CO_2_ seep site at the Yonaguni Knoll IV hydrothermal system, which is characterized by CO_2_ seepage, suggest that habitat segregation of anaerobic bacteria and methanogens occurs in response to differences in CO_2_ concentrations and associated chemical conditions in marine sediments (Inagaki et al., 2006; Konno et al., 2006; Yanagawa et al., 2013). It is also expected that some microbes would be activated by CO_2_ injection under subsurface conditions, because CO_2_ is an important carbon source for autotrophic and mixotrophic microorganisms Previous studies on the activation of microbes associated with Fe^3+^, SO^2−^_4_ reduction and methanogenesis by CO_2_ injection suggest that microbial CO_2_ conversion to available carbon species lead to novel sustainable CO_2_ recycling system (Kirk, 2011; Mayumi et al., 2013).

Although laboratory-based GCS experiments could potentially clarify the impacts of CO_2_ injection on geologic formations, such studies have not yet been attempted. On the other hand, high-pressure incubators have been developed to culture microbes under *in-situ* conditions (Zobell and Oppenheimer, 1950; Yayanos et al., 1979; Orcutt et al., 2008; Sauer et al., 2012) and to simulate subsurface hydrothermal alteration of basaltic rocks (Seyfried and Janecky, 1985). In an attempt to simulate GCS conditions, a high-pressure flow-through reactor system was developed at the Kochi Institute for Core Sample Research, Japan Agency for Marine-Earth Science and Technology (JAMSTEC) in Kochi, Japan, by referring to previous studies on high-pressure instruments.

In this study, we investigated changes in the geophysical features, constituent minerals and microbial community structures in a column comprised of bituminous coal and sand before and after CO_2_ injection under simulating *in-situ* subsurface conditions and discussed various impacts of CO_2_ injection to geologic formations. CO_2_ was supplemented with anaerobic artificial fluids into a coal-sand column, which were sub-sampled after passing through the column. Concentrations and carbon isotope compositions of dissolved gases and volatile organic carbons in the sub-sampled fluids were determined to monitor CO_2_ impact during experiment. Sub-sampled fluids were also incubated to determine microbes survived through a CO_2_ injection experiment. Conventional batch-type cultivation was performed using same bituminous coal sample to investigate the potential for biological carbon conversion in sample pre-coal.

Fresh bituminous coal and associated sandstone were obtained from a subterranean coal mine and used as an analog of a subsurface coal-sand formation. As mentioned above, coal and sandstone are both considered to be well suited for use as CO_2_ repositories. In addition to the recovery of CBM by CO_2_ injection, immature hydrocarbon reservoirs (e.g., oil, bituminous coal and lignite) contain a variety of organic molecules and gases (e.g., H_2_ and CO_2_) generated during maturation of carbonaceous compounds in the hydrocarbon reservoirs accompanied by sedimentation. These compounds, in turn, are utilized by a variety of microorganisms in the nutrient-limited subsurface sediments. Consequently, development of microbial communities that consist of various bacteria, fungi and methanogenic archaea has been reported in these hydrocarbon reservoirs (Fakoussa, 1988, 1990; Edwards and Grbic-Galic, 1994; Nazina et al., 1995; Krüger et al., 2008; Strapoć et al., 2008), suggesting the possibility of biological CO_2_ conversion system (Bio-CCS) that responds to the GCS.

## Materials and methods

### Geobio-reactor system

**Figure 1:**
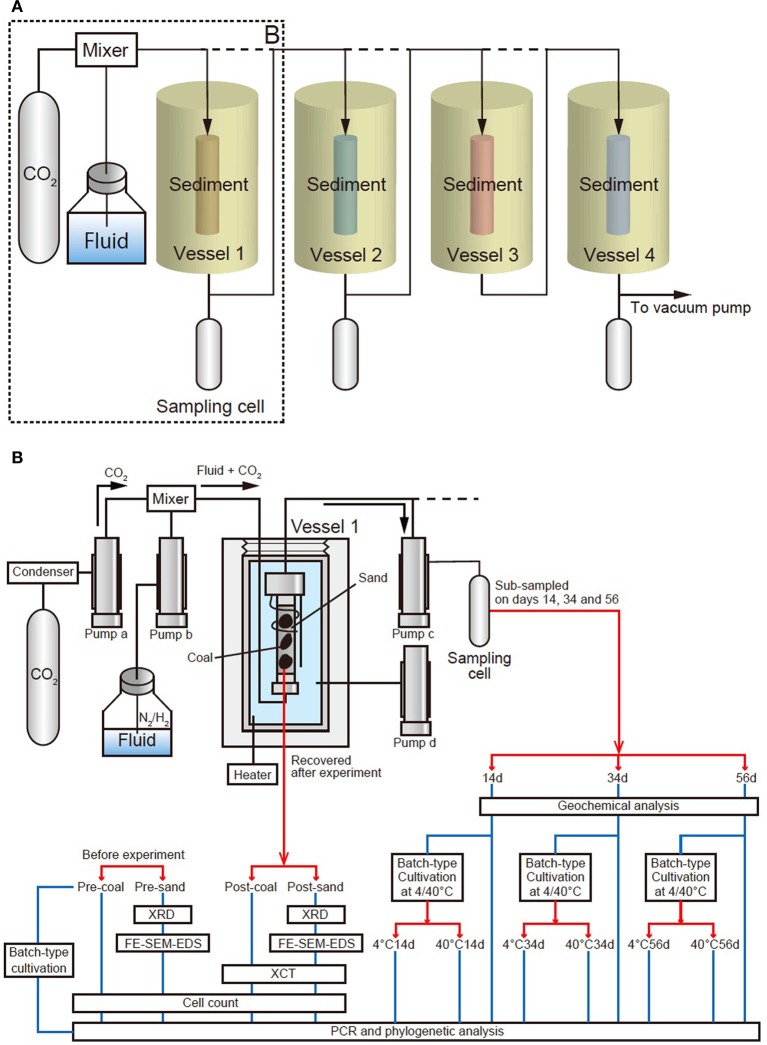
**Schematic diagram of the geobio-reactor system**. The system simulates *in-situ* subsurface pressure and temperature conditions ranging from 0 to 100 MPa and 0–70°C. **(A)** A schematic view of entire the reactor system, comprising four vessels and three sampling cells. CO_2_ and fluid are supplemented into a maximum of four vessels through a mixer. **(B)** Detail of the area dashed box (‘B’) in Figure 1A. Vessel 1 is connected to two upper cylinder pumps which supply CO_2_ and fluid (CO_2_: pump a, fluid: pump b). Another pump below the vessel (pump c) receives CO_2_-injected fluid that has passed through the sediment. Confined pressure is controlled by a cylinder pump d associated with the vessel filled with water (pressure medium), which is disconnected from the main line. CO_2_-injected fluids in pump c were sub-sampled by pressure-keeping sampling cell on days 14, 34, and 56 (Samples 14d, 34d, and 56d, respectively). These fluid samples were injected into tube filled with medium and incubated for 2 weeks at 4 and 40°C (4°C: 4°C14d, 4°C34d and 4°C56d; 40°C: 40°C14d, 40°C34d and 40°C56d). The coal-sand column was recovered and analyzed to determine geophysical/mineralogical characteristics and cell number. Coal and sand before experiment (Sample pre-coal and pre-sand) were also analyzed for comparison in the same way as sample post-coal and post-sand. Batch-type cultivation was performed using pre-coal. PCR and phylogenetic analysis were performed on all samples.

**Figure 2:**
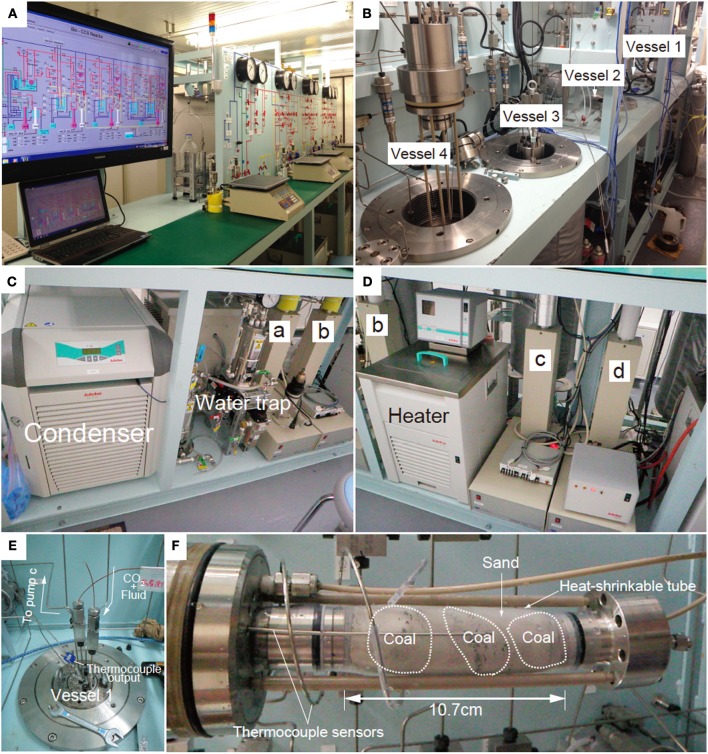
**Photographs of the geobio-reactor system. (A)** Photo of the entire system, **(B)** Photo of the four vessels, **(C)** Cylinder pumps for CO_2_ and fluid supply (pumps a and b). CO_2_ passes through a chiller which keeps the CO_2_ in the liquid phase until it reaches the pump a. **(D)** Cylinder pumps for sub-sampling and controlling confined pressure of the vessel (pumps c and d). **(E)** Photo of the top of the vessel. CO_2_ and fluid are supplemented through the upper line into the vessel. Fluid passing through the sediments is stored in pump c. **(F)** Coal-sand column unit used for CO_2_ injection experiment. Entire length of the column is 10.7 cm and the volume is ca. 81 cm^3^.

The geobio-reactor system consists of four flow-through, high-pressure vessels. The temperature and pressure of these vessels can be independently controlled up to 70°C and 100 MPa using controller (Teledyne Iso, D-series Pump Controller, Nebraska, USA) (Figures 1A, 2A,B). By connecting the four vessels either directly and/or in parallel, the movement of CO_2_ in the subsurface environment, and the associated mineralogical, geochemical and biological reactions that the CO_2_ may be involved in, can be experimentally simulated in the geobio-reactor system. The CO_2_ and/or fluid (e.g., artificial seawater) are supplemented through a mixer into the high-pressure reactor vessels. The pressure conditions and flow rate of the fluid in each vessel (i.e., confined pressure, pore pressure) are regulated by four cylinder pumps (Teledyne Isco, Nebraska, USA, 65D; 68 ml, Figure 1B). CO_2_ and fluid are first introduced into the upper cylinder pumps (pumps a and b, respectively, Figure 1B). The CO_2_ lines from the CO_2_ tank to the cylinder pump are cooled to 5°C by a condenser (F33, Julabo, Baden-Württemberg, Germany) to stabilize liquid CO_2_ phase in the lines (Figures 1B, 2C). The CO_2_ and fluid are then pressurized at the same level and injected into the vessel through a mixer by the cylinder pumps. After passing through the sediment column in the vessel, the CO_2_-injected fluid is stored in the lower pump (pump c; Figures 1B, 2D,E). During reactor operation, the CO_2_-injected fluid can be sub-sampled from a stainless-steel pressure-keeping cell connected to an outlet line from pump C. All of the parameters are displayed and monitored on computers *in-situ* (Figure 2A). All lines are made from Inconel (Special Metals, West Virginia, USA) and separated using several valves at appropriate positions. Waste solution is cleared from lines using a vacuum pump attached to a water trap (Figures 1A, 2C).

Each reaction vessel is constructed from stainless steel. The vessel is filled with a pressure medium (e.g., water) and sediment in the form of a column is inserted from the top. The vessel is then sealed tightly using a thick, threaded stainless-steel lid before conducting each experiment. When conducting an experiment, Mixture fluid of liquid CO_2_ and water is supplemented into the sediment column through polyetheretherketone (PEEK) lines from the bottom part of the column (Figure 1B). Confined pressure is controlled by the cylinder pump (pump d) (Figures 1B, 2D). The pressure medium is circulated through a heater (Julabo, Baden-Württemberg, Germany, HE; up to 70°C) and the vessel to stabilize the temperature in the vessel (Figure 2D). The *in-situ* temperature is monitored by a thermocouple sensor attached to stoppers of the column (Figures 1B, 2E,F).

### CO_2_ injection experiment

CO_2_ injection experiment was performed using only vessel 1 of geobio-reactor system (Figure 1B). All lines of the geobio-reactor were vacuumed before experiment. CO_2_ and fluid were respectively introduced into the upper cylinder pumps (CO_2_: pump a, fluid: pump b). Pore and confined pressure were raised while maintaining the confined pressure higher than pore pressure to prevent fluid leakage from a column. Temperature, pore pressure and confined pressure were set to 40°C, 40 and 41 MPa, respectively. The flow rate of the fluid was 0.002 ml/min. CO_2_ was supplemented into lines from day 14 at 0.00001 ml/min (0.5 vol% vs. fluid) after sampling on day 14. The CO_2_ injection rate was sufficiently lower than the CO_2_ solubility [ca. 8.2 vol%; National Institute of Standards and Technology (NIST); (Duan and Sun, 2003)] to keep the microbes alive in the coal-sand column due to a marked decrease in the pH and destruction of cell structures associated with abundant CO_2_. The experiment was continued for 56 days and total amount of supplied CO_2_ into the coal-sandstone column was 0.81 ml.

The fluids passed through the column were sub-sampled on days 14, 34, and 56 (Sample 14d, 34d, and 56d; Figure 1B) by pressure-keeping sampling cell through pump c (Figure 1B). Dissolved gases and volatile organic carbons in the sub-sampled fluids were analyzed using Gas Chromatography-Isotope Ratio Mass spectrometry (GC-IRMS) and Isotope-Ratio-Monitoring Liquid Chromatography Mass Spectrometry (IRM-LCMS). The samples were also added into sealed test tubes filled by mediums under anaerobic conditions and incubated at 4 and 40°C, respectively. Samples inoculated from a sub-sampled fluid on day 14 and cultivated at 4 and 40°C were named as 4°C14d and 40°C14d. Samples inoculated from a sub-sampled fluid on day 34 and cultivated at 4 and 40°C were named as 4°C34d and 40°C34d. Samples inoculated from a sub-sampled fluid on day 56 and cultivated at 4 and 40°C were named as 4°C56d and 40°C56d (Figure 1B). PCR and phylogenetic analysis was also performed for these fluid samples.

After experiment, coal-sand column was recovered from vessel 1 (Sample post-coal and post-sand, Figure 1B) and analyzed to determine changes of geophysical and mineralogical characteristics using micro X-ray computed tomography (μ XCT scan), X-ray diffraction analysis (XRD), field emission scanning electron microscopy/energy-dispersive X-ray spectrometry (FE-SEM-EDS). Coal and sand were clearly separated based on grain size. Samples post-coal and post-sand were divided into three parts under anaerobic condition for geophysical/mineralogical analysis, cell counts and PCR/phylogenetic analysis. Samples for geophysical/mineralogical analysis were preserved at ambient temperature under aerobic condition. Samples for cell counts were suspended in paraformaldehyde for cell fixation, washed using phosphate buffered saline (PBS) and preserved at −20°C after addition of ethanol (99%). Samples for PCR/phylogenetic analysis were refrigerated at −80°C. Coal and sand before CO_2_ experiment (Sample pre-coal and pre-sand; Figure 1B) were also analyzed for comparison. Conventional batch-type cultivation was also performed using sample pre-coal. All sample names and performed analyses were summarized in Figure 1B. Construction of coal-sand column, composition of artificial fluid in CO_2_ injection experiment, detail of analyses and batch-type cultivations are as follows.

### Construction of coal-sand column

Eocene bituminous coal and sandstone were collected from exposed outcrops in pits 185 m below the ground surface in the Kushiro Coal Mine, Kushiro, Hokkaido on May 31, 2011. The coal layers belong to the Harudori Coal Formation, part of the Urahoro Group comprising a coal bearing formation, sandstone and black shale deposited in the Paleogene period (Hyakkoku, 1966). Collected coal was preserved in an anaerobic bag at 4°C and sandstone was preserved under aerobic conditions at 4°C.

Columned sediment was prepared from chips of Kushiro coal (1–3 cm in diameter) and the coal-bearing sandstone. These samples were enveloped by cylindrical, water-impermeable, heat-shrinkable tube under anaerobic condition as a column (7.5 × 10.7 cm^3^ in volume; Figure 2F). Both ends of the heat-shrinkable tube were sealed using a heat gun to prevent infiltration of the pressure medium from outside.

### Fluid composition

Since the pore water in the coal layer at the Kushiro Coal Mine was almost fresh (Electric conductivity: 1 mS/cm), we determined the composition of the anaerobic artificial fluid by referring to the nutrient medium used for methanogens in fresh anaerobic water (Sakai et al., 2012); specifically, we dissolved the following components into 1 L of Milli Q water (Merck Millipore, Massachusetts, USA): 0.14 g KH_2_PO_4_, 0.54 g NH_4_Cl, 0.24 g MgSO_4_· l,_2_O, 0.13 g CaCl_2_, 0.05 g NaCl, 2 g BD BBL™ yeast extract (BD, New Jersey, USA), 2.5 g NaHCO_3_, 10 ml methanol (50% v/v), 0.01 g Na-acetate, 0.2 g Na-formate, 0.001 g resazurin, 15 ml vitamin solution (4.9 mg biotin, 8.8 mg folic acid, 4.1 mg pyridoxine· HCl, 6.7 mg thiamine· HCl, 7.5 mg riboflavin, 2.4 mg nicotinic acid, 9.5 mg D-pantothenate (Calcium salt), 0.1 mg vitamin B12, 2.7 mg p-aminobenzoic acid and 4.1 mg lipoic acid per litre of Milli Q H2O) and 1 ml trace element solution (1.27 g FeCl2, 0.198 g MnCl2·4H2O, 0.136 g ZnCl2, 0.0062 g H3BO3, 0.025 g NiCl2·6H2O, 0.0133 g AlCl3, 0.001 g NaMoO4· 2H2O, 0.0017 g NaSeO3, 0.0003 g NaWO4·2H2O and 0.0013 g CuCl2 per litre of Milli Q H2O). Resazurin was added to the fluids as a redox indicator to monitor anaerobic conditions in the artificial fluid. The compositions of the vitamin and trace element solutions were a slight modification of Widdel's medium (Widdel, 1986). The pH of the fluid was adjusted to 7. Na_2_S (5 wt%, pH adjusted to 7) was added to the artificial fluid to maintain anaerobic conditions. After anaerobic treatment, the gas phase of the bottled fluid was replaced with N_2_ and the bottles were sealed tightly with butyl rubber stoppers and screw caps. The headspace of the bottle was filled with N_2_/H_2_ (80/20 [v/v]) before removed into geobio-reactor-system.

### Determination of porosities and constituent minerals of coal-sand column

Samples post-coal and post-sand (ca. 1 cm in diameter) were roughly crushed and scanned by μ XCT (HMX225 Microfocus X-ray CT scanner, TESCO Corporation, Kanagawa, Japan) into ~1 mm^3^-size to estimate the porosity of the coal and sand after the experiment. The porosities of sample pre-sand and pre-coal could not be measured due to their inherently brittle properties. Samples pre-sand and post-sand were finely powdered and analyzed by XRD (XRD Phillips X'pert PRO, PANalycal, Almelo, Netherlands) to determine constituent minerals. Detailed chemical changes of constituent minerals were observed by FE-SEM-EDS (JEOL, JSM-6500F, Tokyo, Japan). Samples for FE-SEM-EDS observation were crushed into course powder and fixed on carbon tape of the sample stage.

### Cell count

Cells were separated from suspensions of samples pre-coal, pre-sand, post-coal and post-sand by heavy-solution separation (Nycodenz 30, 50, 80% [w/v] [1.159, 1.265 and 1.426 g/ml] and Na6H2W12O40· H2O 65% [w/w]) and concentrated to accumulate sufficient cells for counting, respectively (Morono et al., 2013). Concentrated cells were filtrated through Isopore™ membrane filters (0.2 μm GTBP, Merck Millipore, Massachusetts, USA) and stained with a fluorescent dye (SYBR Green, Takara Bio, Kyoto, Japan). Cells on membrane filters were then counted by observation under a fluorescence microscope (Olympus BX51Wl, Olympus, Tokyo, Japan) (Morono et al., 2009).

### Batch-type cultivation of sub-sampled fluids

Sub-sampled fluids (Samples 14d, 34d, and 56d) were recovered and preserved in sealed tubes under anaerobic condition. The presence of microbes in these samples was confirmed by observation under an optical microscope (Olympus BX51Wl, Olympus, Tokyo, Japan). Then, 5 ml medium was prepared in 15 ml tubes and inoculated with 1 ml samples 14d, 34d, and 56d under anaerobic conditions, respectively. Composition of the medium was the same as artificial fluids used in CO_2_ injection experiment. After inoculation, the tubes were incubated at 4 and 40°C for 2 weeks under an atmosphere of N_2_/CO_2_ (80/20 [v/v]).

### DNA extraction of coal and sand

DNA extraction was performed using samples of pre-coal, pre-sand, post-coal and post-sand. DNA was manually extracted from approximately 10 g of the ground samples using a hot alkaline extraction method (Morono et al., in preparation). Briefly, the sample was pre-treated with alkaline lysis solution consisting 1M NaOH, 5 mM EDTA (pH 8.0), and 1% SDS at 70°C for 20 min, and then DNA was recovered by phonol:chloroform:isoamyl alcohol (25:24:1) and chloroform:isoamyl alcohol (24:1). The extracted DNA was then further purified and concentrated by NucleoSpin Gel and PCR Clean-up kit (Macherey-Nagel, Düren, Germany) followed by ethanol precipitation.

### PCR amplification and sequencing of bacterial and archaeal 16s rRNA genes

PCR amplification of bacterial and archaeal 16S rRNA genes was performed using SYBR premix ExTaq (Takara Bio, Kyoto, Japan) with a StepOnePlus real-time PCR system (Applied Biosystems, Foster City, CA, USA) after determining the optimum number of PCR cycles using multiple primer sets (Table 1). Of the primers tested, only 27F/338R, 341F/926R and 806F/958R could amplify the 16S rRNA gene with an annealing temperature of 56°C. The number of PCR cycles was 32 and 42 cycles for bacteria and archaea, respectively. The PCR products were amplified with the primer sets 341F/926R and 806F/958R and purified using a NucleoSpin Gel and PCR Clean-up kit (Macherey-Nagel, Düren, Germany), and the purified products were cloned into pCR2.1 TOPO vector (Invitrogen, Carlsbad, CA, USA) according to the manufacturer's instruction. The recombinant vector was transformed into Ecos competent *Escherichia coli* DH5α cells (Nippon Gene, Tokyo, Japan). The sequences of the inserts were determined using an ABI3130xl Genetic Analyzer (Applied Biosystems, Foster City, CA, USA) and the obtained 16S rRNA gene sequences were classified using Mothur Utility package (Schloss et al., 2009). The 16S rRNA gene sequences reported in this study were deposited in the GenBank/EMBL/DDBJ database under accession numbers AB40305 to AB40587. In this study, samples pre14d, pre34d and pre56d in the GenBank/EMBL/DDBJ database were renamed as 14d, 34d and 56d, respectively.

**Table 1:** **List of the primer sets for PCR**.

**Taget gene**	**Primer name**	**Sequence (5′ to 3′)**	**Paired primer**	**References**
16S rRNA gene of *Bacteria*	27F	AGA GTT TGA TCC TGG CTC AG	338R, 926R, 1490R	Edwards et al., 1989
	338R	GCT GCC TCC CGT AGG AGT	27F	Suzuki and Giovannoni, 1996
	341F	CCT ACG GGA GGC AGC AG	926R	Muyzer et al., 1998
	926R	CCG TCA ATT CCT TTR AGT TT	27F, 341F	Muyzer et al., 1995
	21F	TTC CGG TTG ATC CYG CCG GA	921R, 958R, 1490R	DeLong, 1992
16S rRNA gene of *Archaea*	806F	ATT AGA TAC CCS BGT AGT CC	958R	Takai and Horikoshi, 2000
	912R	CCC CCG CCA ATT CCT TTA A	21F	Miyashita et al., 2009
	958R	YCC GGC GTT GAM TCC AAT T	21F, 806F	DeLong, 1992
16S rRNA gene of *Bacteria and Archaea*	1490R	GGH TAC CTT GTT ACG ACT T	27F, 21F	Weisburg et al., 1991
mcrA gene	Me1F	GCM ATG CAR ATH GGW ATG TC	Me2R	Hales et al., 1996
	Me2R	TCA TKG CRT AGT TDG GRT AGT	Me1F	Hales et al., 1996
	mcrAimprov_F	TWY GAC CAR ATM TGG YT	mcrAimprov_R	Yanagawa et al., 2011
	mcrAimprov_R	ACR TTC ATB GCR TAR TT	mcrAimprov_F	Yanagawa et al., 2011

### PCR amplification of mcrA gene

Detection of the methyl co-enzyme M reductase (*mcrA*) gene was attempted using two primer sets (Table 1). The PCR consisted of 50 cycles and an annealing temperature of 54°C. In order to increase the detection sensitivity, multiple displacement amplification (MDA) was performed using Phi29 DNA polymerase (New England Biolabs, Beverly, MA, USA) according to the manufacturer's instructions. The amplification was performed at 30°C for 29 h. MDA products were purified using an Amicon Ultra-0.5 ml 30 K filter unit (Millipore, Bedford, MA, USA). Subsequently, the *mcrA* gene in the MDA product was amplified by PCR as described above.

### Batch-type cultivation for enrichment of methanogens

To cultivate methanogens from sample pre-coal, batch-type cultivation was performed in 500 ml bottles containing 300 ml media under an atmosphere of N_2_/CO_2_ (80/20 [v/v]) without shaking (Figure 7). The basal medium was the same medium used for the geobio-reactor system without organic compounds and contained the following energy sources: (i) approximately 100 kPa H_2_ (in the head space) and 20 mM formate to obtain hydrogenotrophic methanogens; (ii) 10 mM acetate to enrich aceticlastic methanogens; and (iii) 5 mM methanol and 5 mM trimethylamine to cultivate methylotrophic methanogens. We also added coenzyme M (2-mercaptoethanesulfonic acid) to the medium at a final concentration of 0.5 mM, because the growth of some methanogens is stimulated/required by coenzyme M (e.g., Bräuer et al., 2011). For the hydrogen plus formate culture, acetate was also added as a carbon source at a final concentration of 1 mM. The coal samples were crushed using a sterilized hummer and then placed in a bottle containing the anaerobic medium. The bottles were then tightly closed with butyl rubber stoppers and screw caps and strictly anaerobic conditions were created by the addition of reducing agents. All of the cultures were incubated at 20°C. Separate culture bottles were used for each substrate condition. To monitor anaerobic conditions in the media, resazurin was added to the medium as a redox indicator. Methane concentration was determined by gas chromatography (GC3200G, GL Science) with a thermal conductivity detector. An Olympus microscope (Olympus BX51F, Olympus, Tokyo, Japan) with a colour CCD camera system (Olympus DP72) was used to examine cell morphology and epifluorescence.

### Nucleic acid extraction, PCR and phylogenetic analysis for methanogens

Total DNA extraction, PCR amplification and sequencing were performed as described previously (Miyashita et al., 2009). For PCR amplification, primers 21F and r912R were used to obtain the archaeal 16S rRNA gene sequence of methanogens in the coal enrichment cultures. Phylogenetic analysis using 16S rRNA gene sequences was performed with the ARB program (Ludwig et al., 2004). A 16S rRNA gene-based tree was constructed using the neighbour-joining method for sequences >1000 nucleotides. Shorter sequences (i.e., sequences obtained in this study) were inserted into this tree without changing the tree topology by using the parsimony insertion tool of the ARB program (Ludwig et al., 2004). Bootstrap analysis was performed with the MEGA 5 program (Tamura et al., 2011) for 1000 resamplings to estimate the confidence of the 16S rRNA gene tree topology. The 16S rRNA gene sequences reported in this study were deposited in the GenBank/EMBL/DDBJ database under accession numbers AB828144 and AB828145.

### Geochemical analysis

To determine concentrations and isotopic compositions of dissolved gases in sub-sampled fluids (Sample 14d, 34d and 56d), we extracted the dissolved gases in the fluid under vacuum (Saegusa et al., 2006). The gas extraction procedure was as follows: 28.5–39 ml of the sub-sampled fluids was recovered in a gas-tight cylinder and transferred to a pre-evacuated glass extraction bottle (~370 ml), leaving 331–341.5 ml headspace in the extraction bottle. In order to facilitate the extraction of the dissolved gases from the fluid into the headspace, the extraction bottle was ultrasonicated at 25°C for 5 min. To determine the total gas volume in the fluid, the pressure was measured by a pressure gauge. The extracted headspace gas was then sub-sampled into vacuumed glass vials. After extraction of dissolved gases, the fluid was further processed for the analysis of dissolved components, dissolved inorganic carbon (DIC), formate, acetate, and methanol.

CBM in pre-coal was extracted to determine carbon isotopic composition of CH_4_ (δ^13^C_CH4_). For the extraction, ca. 3 cm^3^ of coal fragments of pre-coal were introduced into a 21.5 ml vial, which was filled with 5 ml Milli Q water sealed with a butyl stopper and crimp capped. The vial was shaken by vortexing and then heated in an oven at 70°C for 2 h. Headspace gas (~0.5 ml) was then extracted with a gas-tight syringe for the isotopic analysis.

H_2_, CH_4_, and CO_2_ concentrations in the extracted gas from the sub-sampled fluids were analyzed by gas chromatography with a He ionization detector (HID) using a SRI 8610C (SRI Instruments, California, USA). The standard deviation obtained for repeated analysis of the laboratory standard gas was <4%. Carbon isotopic composition of CH_4_ (δ^13^C_CH4_) was determined by gas chromatography isotope-ratio-mass spectrometry (GC-IRMS; Thermo-Finnigan Delta Plus XP isotope-ratio mass spectrometer connected to TRACE GC and GC-COMBUSTION III). DIC concentration and δ^13^C_DIC_ in sub-sampled fluids were measured with a ThermoFinnigan Delta Plus XP IRMS instrument connected to Thermo Scientific TC/EA via a ConFlo III interface in a similar way to that described by Miyajima et al. (1995) and Toki et al. (2004). The standard deviation obtained for repeated carbon isotope analysis of the laboratory standard (NaHCO_3_ solution) was <0.2‰. We calculated the ΣCO_2_ concentrations by adding the DIC concentrations with CO_2_ concentrations.

The concentrations and δ^13^C of formate, acetate, and methanol were determined by Isotope-Ratio-Monitoring Liquid Chromatography Mass Spectrometry (IRM-LCMS); Thermo-Finnigan Delta Plus XP isotope-ratio mass spectrometer connected to LC IsoLink), as described by Heuer et al. (2006) and Ijiri et al. (2012).

### Thermodynamic calculation

To study theoretical constraints for the habitability of bacteria and methanogens, we calculated Gibbs free energies (ΔG) of a possible microbial reaction using *in-vitro* geochemical data obtained in the present study. Enthalpy (ΔH°), standard Gibbs free energy (ΔG°) (25°C, 1 atm) and Gibbs free energy at 40°C and 41 MPa at equilibrium (ΔG°_40,41_) were estimated using the SUPCRT92 database (Shock and Helgeson, 1990; Johnson et al., 1992). ΔG at 40°C and 41 MPa was calculated using as following reaction equation: ΔG_40,41_ = ΔG°_40,41_ – RTlnQ, where R is the universal gas constant 8.31 J· K^−1^· 1wh^−1^, T is the temperature in kelvin, and Q is the reaction quotient. Activity coefficients of all dissolved chemical species involved in the calculation of Q were estimated from geochemical data using Spec E8 of the Geochemist's Workbench 7.0 (summarized in Table 5).

## Results

### Changes of physical and chemical properties of coal-sand column

The redox state of fluid was continuously monitored by the color of resazurin, showing that anaerobic condition was successfully maintained during the geobio-reactor operation and subsampling. The total and effective porosity of post-coal and post-sand are shown in Table 2. Post-sand showed a high total and effective porosity, which is much higher than that of post-coal. These results suggest that pore water could pass all the way through the column without considerable stress since the fine coal was entirely enveloped by sand in the heat-shrinkable tube (Figure 2F).

**Table 2:** **Porosities and cell numbers in pre-coal, pre-sand, post-coal, and post-sand**.

**Sample**	**Total porosity (vol.%)**	**Effective porosity (vol.%)**	**Cell abundance (10^3^ cells/cm^3^**
Pre-coal	NA	NA	10.3
Pre-sand	2.405	0.958	9.2
Post-coal	NA	NA	6.8
Post-sand	2.489	2.476	3.7

*Not available*.

**Figure 3:**
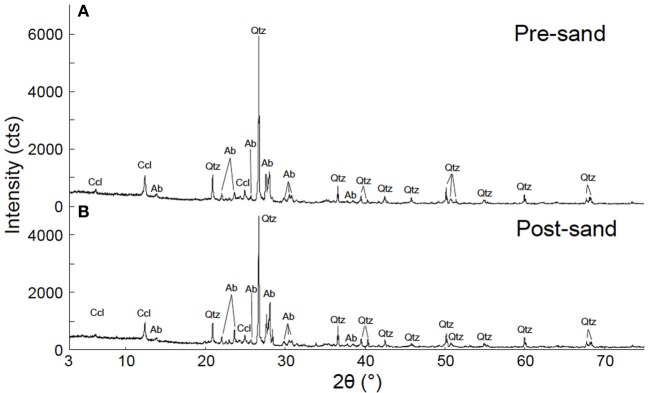
**Result of XRD analysis. (A)** XRD pattern of pre-sand. **(B)** XRD pattern of post-sand. Qtz, quartz; Ab, albite; Ccl, clinochlore.

**Figure 4:**
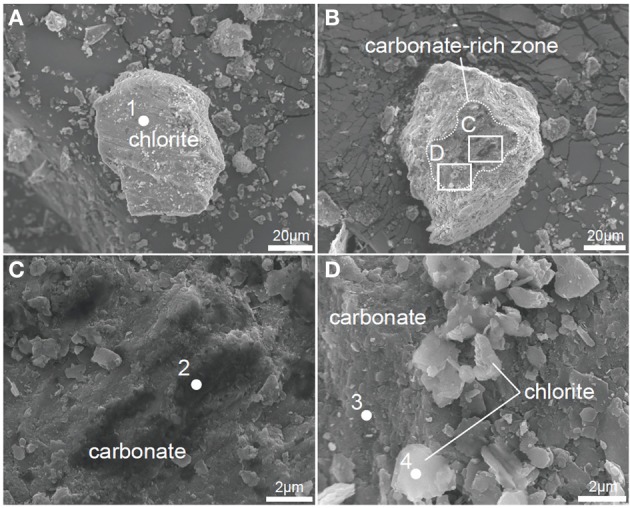
**FE-SEM images of powdered pre-sand and post-sand. (A)** Chlorite in pre-sand. **(B)** Carbonated chlorite aggregate in post-sand. **(C)** Magnified view of inset in **(B)** to show carbonate and chlorite aggregate in post-sand, respectively. **(D)** Magnified view of inset in **(B)** to show chlorite grains and the carbonated part of the chlorite aggregate.

XRD analysis suggests that post-sand comprises quartz, albite and chlorite (clinochlore), and has a similar volume ratio of minerals to that of pre-sand (Figure 3). On the other hand, FE-SEM-EDS observations of post-sand revealed the existence of minor minerals containing high amounts of C, Ca, Mg, and Fe as the major elements, and Si and Al as the minor elements (measurement points 2 and 3 in Table 3 and Figures 4B–D), while chlorite aggregates are dominant in pre- and post-sand (measurement points 1 and 4 in Table 3, Figures 4A,D). Considering the absence of major anion species except CO^2−^_3_, the minor minerals are most likely carbonates. These results show that carbonation of the surface of chlorite aggregates proceeded slowly in post-sand and that no carbonate existed in pre-sand.

**Table 3:** **Chemical compositions of representative carbonate and chlorite in samples pre-sand and post-sand**.

**Sample**	**Pre-sand**	**Post-sand**		
	**1**	**2**	**3**	**4**
**Mass %**				
C	3.9	34.5	10.7	8.7
O	48.8	27.1	49.7	57.2
F	<d.I.	0.1	0.3	0.1
Na	6.2	0.2	<d.I.	<d.I.
Mg	<d.I.	5.6	6.5	2.0
Al	11.3	0.4	1.3	10.6
Si	27.1	1.8	2.8	14.0
K	<d.I.	0.6	0.5	0.4
Ca	2.7	21.8	21.0	5.2
Fe	<d.I.	7.9	7.2	1.9
Total	100.0	100.0	100.0	100.0

*<d.I.: below detection limit of energy-dispersive X-ray spectrometry (EDS)*.

### Changes of microbial communities in coal-sand column and sub-sampled fluids

**Figure 5:**
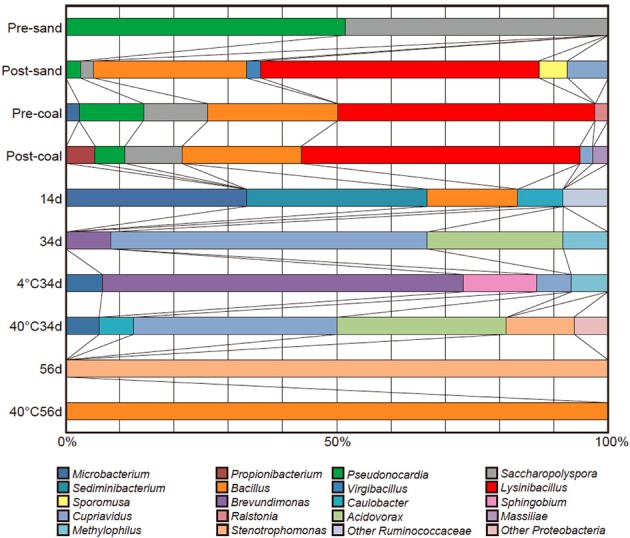
**Cloning analyses of pre-sand, post-sand, pre-coal, post-coal, sup-sampled fluids (Samples 14d, 34d, and 56d), and the cultivated samples (Samples 4°C34d, 40°C34d and 40°C56d)**.

The microbial community in pre-coal was dominated by *Lysinibacillus* and *Bacillus*, which was similar to the community structure in post-coal (Figure 5). Aerobic bacteria (e.g., *Saccharopolyspora* and *Pseudonocardia*) inhabited pre-sand because they were preserved in aerobic condition after sampling from Kushiro Coal Mine. The microbial community in post-sand was similar to that in pre- and post-coal, suggesting that bacteria in pre-coal migrated to surrounding sand together with the CO_2_-injected fluid during the experiment. Compared to pre-coal and pre-sand, cell numbers decreased slightly in post-coal and post-sand (Table 2). Given the high porosity of sand, cells in the coal-sand column were transported by the CO_2_-injected fluid.

**Figure 6:**
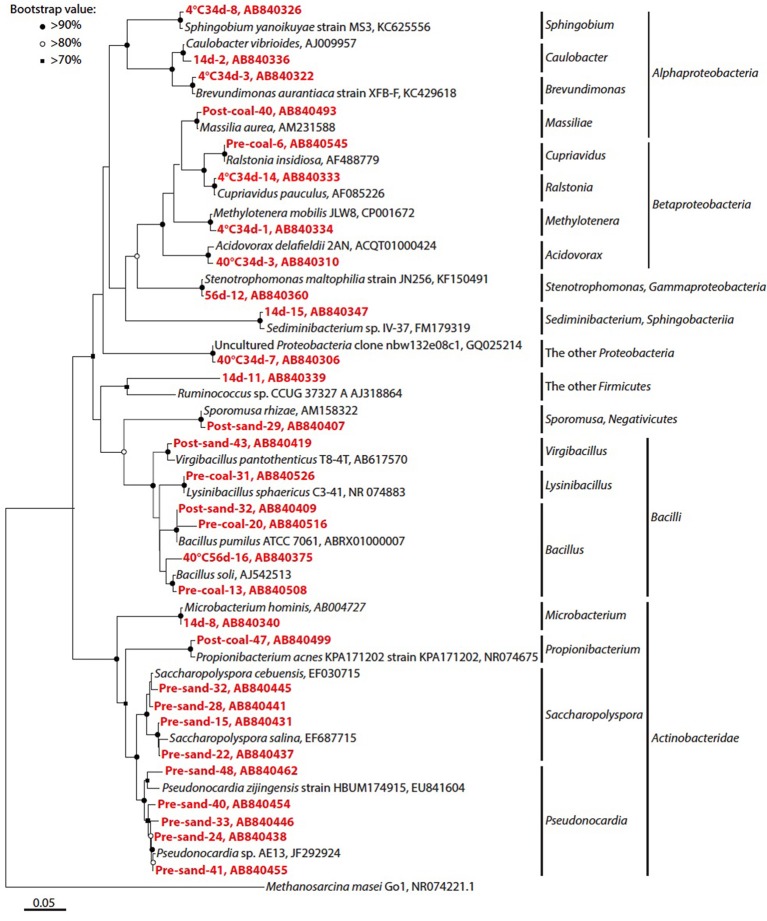
**Phylogenetic analysis of bacterial 16S rRNA gene sequences**. Only representative sequences from each operational taxonomic unit (OTU) at a 97% cut-off were presented. Accession numbers are also shown after each strain or clone name. Red colored number prefixed the each clone name indicate the clone number of each sample. Neighbor-joining tree was constructed using the MEGA 5.10 software (Tamura et al., 2011). Bootstrap values (>60%) obtained after 1000 iterations are indicated at nodes.

Microbial growth was confirmed in sub-sampled fluids (Samples 14d, 34d, and 56d) and the cultivated samples (Samples 4°C34d, 40°C34d, and 40°C56d). Cloning analysis was also performed on these samples to identify the microbial community in the coal-sand column (Figure 5; Table 4). *Bacillus, Cupriavidus*, and *Microbacterium* were observed with sequencing in the post-coal and post-sand, as well as in the sub-sampled fluids and the cultivated samples; however, the community structure of these samples differed markedly from each other (Figure 5). In sample 14d, *Sediminibacterium*, *Microbacterium* and *Bacillus* were dominant. Microbial communities in 34d, as well as samples 4°C34d and 40°C34d were relatively similar and were dominated by *Cupriavidus* and *Brevundimonas*. Bacterial diversity decreased dramatically in sample 56d and in 40°C56d. No bacterial growth was observed in 4°C14d and 40°C14d, or in 4°C56d. Archaeal clones belonging to South African Gold Mine Euryarchaeotic Group (SAGMEG), Gulf of Mexico arc I group (GOM arc I) and Misscelaneous Crenarchaeotal Group (MCG) were obtained by means of 42 cycles for PCR from the samples of pre-coal, post-coal, post-samd and 40°C34d (data is not shown, accession number AB840549-AB840587). The PCR amplification of archaeal 16S rRNA genes required higher number of the reaction cycle than those for bacteria (i.e., 32 cycles), indicating that bacteria dominate microbial communities we examined and the archaeal components are relatively minor. In archaeal clone libraries, no known methanogenic 16S rRNA sequence and *mcrA* gene were detected. Phylogenetic tree of the bacterial 16S rRNA gene sequences obtained from all analyzed samples were shown in Figure 6.

**Table 4:** **Results of cloning analysis of bacteria in samples pre-sand, post-sand, pre-coal, post-coal, 14d, 34d, 4C34d, 40C34d, 56d, and 40C56d**.

**Genus**	**Pre-sand**	**Post-sand**	**Pre-coal**	**Post-coal**	**14d**	**34d**	**4C34d**	**40C34d**	**56d**	**40c56d**
*Microbacterium*	0	0	1	0	4	0	1	1	0	0
*Propionibacterium*	0	0	0	2	0	0	0	0	0	0
*Pseudonocardia*	19	1	5	2	0	0	0	0	0	0
*Saccharopolyspora*	18	1	5	4	0	0	0	0	0	0
*Sediminibacterium*	0	0	0	0	4	0	0	0	0	0
*Bacillius*	0	11	10	8	2	0	0	0	0	15
*Virgibacillus*	0	1	0	0	0	0	0	0	0	0
*Lysinibacillus*	0	20	20	19	0	0	0	0	0	0
*Sporomusa*	0	2	0	0	0	0	0	0	0	0
*Brevundimonas*	0	0	0	0	0	1	10	0	0	0
*Caulobacter*	0	0	0	0	1	0	0	1	0	0
*Sphingobium*	0	0	0	0	0	0	2	0	0	0
*Cupriavidus*	0	0	0	1	0	7	1	6	0	0
*Ralstonia*	0	0	1	0	0	0	0	0	0	0
*Acidovorax*	0	0	0	0	0	3	0	5	0	0
*Massilia*	0	0	0	1	0	0	0	0	0	0
*Methylophillus*	0	0	0	0	0	1	1	0	0	0
*Stenotrophomonas*	0	0	0	0	0	0	0	2	12	0
*Other Ruminococcaceae*	0	0	0	0	1	0	0	0	0	0
*Other Proteobacteria*	0	0	0	0	0	0	0	1	0	0
Total clone number	37	39	42	37	12	12	15	16	12	15

### Cultivation of methanogens from sample pre-coal

**Figure 7:**
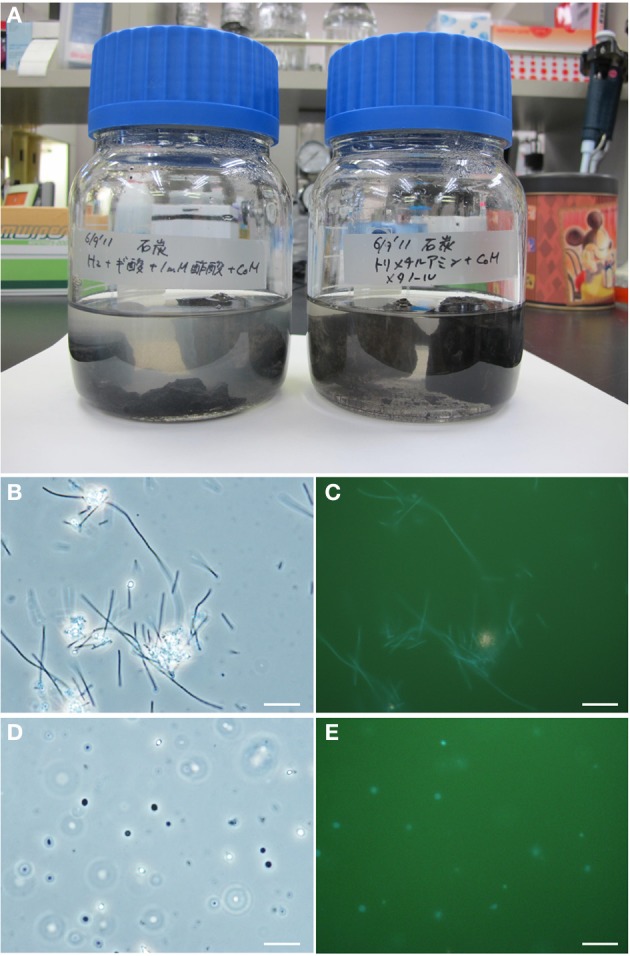
**Micrographs of methanogenic enrichment cultures established from the coal sample. (A)** Culture bottles. **(B–E)** Photomicrographs of the enrichment cultures. Phase-contrast micrographs **(B,D)** and fluorescence micrographs **(C,E)** of the same fields are shown. **(B,C)** Rod-shaped methanogen cells grown on H_2_/CO_2_-formate medium; and **(D,E)** coccoid-shaped methanogen cells grown on methanol-trimethylamine medium. Bars represent 10 μm.

**Figure 8:**
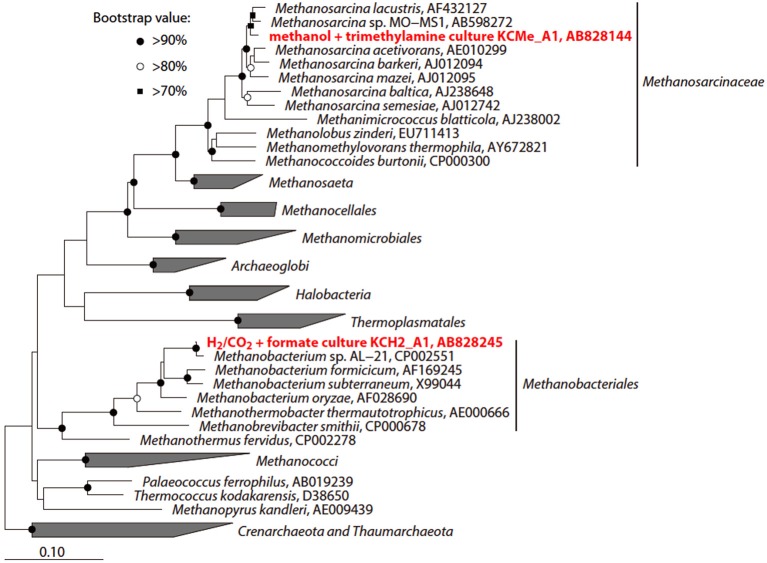
**Phylogenetic tree showing the position of 16S rRNA gene sequences obtained from the methanogenic enrichment cultures**. The initial tree was constructed with sequences greater than 1000 nucleotides using the neighbor-joining method. Subsequently, the sequences obtained in this study were inserted into the tree by the using the parsimony insertion tool of the ARB program. Accession numbers are also shown after each strain or sequence name. The scale bar indicates the estimated number of base changes per nucleotide position. The symbols at the branch nodes show the bootstrap values (>70%) obtained after 1000 resamplings.

To investigate the potential for biological CO_2_ reduction to methane in Kushiro coal, we anaerobically incubated sample pre-coal using conventional batch-type cultivation technique in a medium for methanogens (Figure 7A). We prepared three cultures; i.e., H_2_ + formate-fed, acetate-fed, and methanol + trimethylamine-fed cultures, based on the three major groups of methanogens, i.e., hydrogenotrophic, acetoclastic, and methylotrophic methanogens (Liu and Whitman, 2008). After 1 year of incubation at 20°C, methane was detected in all of the cultures except the acetate-fed culture. Microscopic observations showed that almost all the cells in these methane-producing cultures consisted of F_420_-autofluorescence methanogen-like cells. Rod-shaped and coccoid-shaped cells having F_420_-like autofluorescence were dominant in the H_2_ + formate-fed and methanol-trimethylamine-fed cultures, respectively (Figures 7C,E). To identify the methanogens in the cultures, we sequenced archaeal 16S rRNA genes amplified by PCR using a universal archaeal primer set; 16S rRNA gene-based clone analysis was not used because the methanogen-like cells in each culture exhibited the same morphology. The sequence obtained from the H_2_ + formate-fed culture was affiliated with the hydrogenotrophic methanogen genus *Methanobacterium*. The archaeal 16S rRNA gene sequence obtained for the methanol-trimethylamine culture belonged to the methylotrophic methanogen genus *Methanosarcina*. These sequences had high sequence similarities (>99%) to known methanogens (Figure 8). For the acetate-fed culture, no cell proliferation or methane-production was observed after 2 years of incubation.

### Dissolved gas compositions and volatile organic carbons

**Figure 9:**
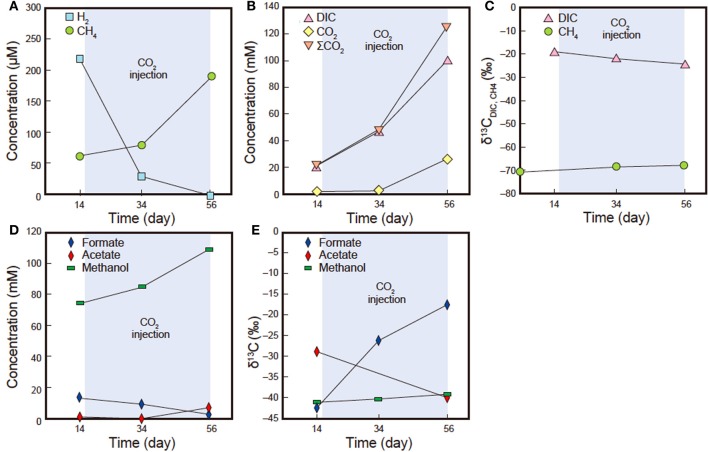
**H_2_, CH_4_, CO_2_, DIC, ΣCO_2_, formate, acetate, methanol concentrations and carbon isotope compositions (δ^13^C) of DIC and CH_4_ insub-sampled fluids (samples Pre14d, Pre34d and Pre56d). (A)** Dissolved H_2_ and CH_4_ concentrations. **(B)** Dissolved CO_2_, DIC and ΣCO_2_ concentrations. **(C)** δ^13^C_DIC_ and δ^13^C_CH4_. **(D)** Dissolved formate, acetate and methanol concentrations. **(E)** δ^13^C_formate_, δ^13^C_acetate_ and δ^13^C_methanol_. Standard deviation obtained after repeated analysis of the laboratory standard gas was <4%. Standard deviation obtained from repeated carbon isotope analysis of the laboratory standard (NaHCO_3_ solution) was <0.2‰.

The geochemical characteristics of the sub-sampled fluids are summarized in Table 5. Over the course of the experiment, the dissolved CH_4_ concentrations increased (63.6–186 μ M) while δ^13^C_CH4_ remained constant (−68.7 to −68.3‰, Figures 9A,C). The δ^13^C_CBM_ value of sample pre-coal was −71.4‰ at day 0 (Figure 9C), which was similar to the dissolved δ^13^C_CH4_ value in all of the sub-sampled fluids. Dissolved CO_2_, DIC and ΣCO_2_ increased during the experiment, with ΣCO_2_ (22.1–125.6 mM) consistently lower than expected amount of ΣCO_2_ (initial concentration: 138.4 mM) in all samples (Figure 9B). δ^13^C_DIC_ was depleted during experiment (−19.4 to −24.4‰). Dissolved H_2_ decreased from 217.2 to 0 μ M between days 14 and 56 (Figure 9A). An increase in the acetate concentration was observed (0.8–7.0 mM), whereas δ^13^C_acetate_ was depleted during experiment (−28.9 to −40.1‰) (Figures 9D,E). On the other hand, the formate concentration decreased and δ^13^C_formate_ was enriched in ^13^C. In addition, the methanol concentration also increased, while the δ^13^C_methanol_ values remained constant for the duration of the experiment.

**Table 5:** **Geochemical data of samples 14d, 34d, and 56d**.

**Sample name**	**14d**	**34d**	**56d**
**Sampling time (day)**	**14th**	**34th**	**56th**
H_2_(μM)	217.2	30.8	0.0
CH_4_ (μM)	63.6	79.7	186.1
CO_2_ (μM)	1.2	2.2	26.1
DIC (mM)	20.9	46.6	99.5
∑CO_2_(mM)	22.1	48.8	125.6
Formate (mM)	13.2	9.1	2.6
Acetate (mM)	0.8	<0.075	7.0
‰Methanol (mM)	74.1	84.8	109.0
δ^13^ C-CH_4_ (‰ VPDB)	−71.4^*^	−68.7	−68.3
δ^13^ C-DIC (‰ VPDB)	−24.4	−21.9	−19.2
δ^13^ C-formate (‰ VPDB)	−42.6	−26.2	−17.7
δ^13^ C-acetate (‰ VPDB)	−28.9	NA	−40.1
δ^13^ C-acetate (‰ VPDB)	−41.0	−40.1	−38.8

* *Coal-bed CH_4_ from pre-coal*.

*NA, not available*.

## Discussion

### Migration and pressure tolerance of bacteria

Cloning analysis of samples pre-coal, pre-sand, post-coal and post-sand revealed that bacteria migrated from coal to surrounding sand. Furthermore, some of these bacteria were moved to the sub-sampling cell by CO_2_-containing fluid due to the high porosity of the sand. The mechanisms of microbial migration have been investigated in a variety of natural environments, either for purposes of bioremediation or to prevent microbial contamination (e.g., drinking water pumped from an aquifer) (Ferguson et al., 2003; Smith and Perdek, 2004). Numerous laboratory experiments and field observations of microbial migration have been examined using various mathematical models (Harvey et al., 1995; Johnson et al., 2001; Harvey and Harms, 2002; Tufenkji, 2007). However, the change of microbial communities associated with migration is still poorly understood. Cloning analyses of sub-sampled fluids and the cultivated samples showed that, with the exception of a few genera, the microbial communities differed markedly from those in the coal-sand column. These results can be explained by two sorting events of bacterial communities as follows: microbial effluence to CO_2_-injected fluid from the coal-sand column and decompression after sample recovery. The coal-sand column would retain some bacteria and prevent them from being transported in the flow of CO_2_-injected fluids. Cultivation experiments using sub-sampled fluids revealed that *Bacillus, Cupriavidus*, and *Microbacterium* were capable of surviving pressurization/decompression, suggesting that certain terrestrial bacteria in coal-sand formations have the ability to resist marked changes in pressure. Furthermore, it is conceivable that spore formation might be a possible function to survive drastic environmental changes associated with the GCS.

Although anaerobic conditions were maintained in all lines and vessels, aerobic *Lysinibacillus* bacteria were dominant in the microbial community of coal-sand column. It is possible that *Lysinibacillus* was able to survive the anaerobic conditions in coal-sand column by producing spores. Indeed, *Lysinibacillus* was not observed in any of the fluid samples collected, suggesting that *Lysinibacillus* does not produce spores under ambient pressure conditions or they could not migrate with fluid flow and remained in the column.

Microbial migration associated with fluid flow is expected to occur in natural subsurface formations if the formation has enough pore-throat connectivity. In addition, it is considered that artificial CO_2_ injected will positively enhance microbial migration. Thus, considering that the microbial communities in coal and sand became similar after experiment, it is possible that bacterial communities may be very homogeneous in porous subsurface coal-sand formations used as geological CO_2_ repositories.

### Origin of dissolved CH_4_

The relationship between δ^13^C_CH4_ and δ^13^C_DIC_ is good indicator of the biogenic production of CH_4_ during experiment (e.g., Whiticar, 1999). When the CH_4_ is produced by hydrogenotrophic methanogenesis or acetoclastic methanogenesis during experiment, the δ^13^C_CH4_ value should be affected by the δ^13^C values of precursors of CH_4_ such as δ^13^C_DIC_ and δ^13^C_acetate_. However, the δ^13^C_CH4_ remained constant through the experiment and was similar to δ^13^C_CBM_, even though the δ^13^C_DIC_ and δ^13^C_acetate_ were depleted during the experiment. Those carbon isotopic compositions suggest that the CH_4_ dissolved in fluids not originated from biogenic but from CBM in pre-coal. Biogenic CH_4_ was not detected likely because of the slow metabolic rate or inactivation of methanogens in the experimental condition. It is most likely that CH_4_ discharge was enhanced by the CO_2_-injected fluid that was supplemented into the coal-sand column as previously reported (Gunter et al., 1997; White et al., 2005). Indeed, the results of the PCR and cloning analysis showed that known methanogen was not observed in any examined samples, which is consistent with the lack of any biogenic changes in δ^13^C_CH4_ values.

### H_2_, CO_2_, acetate, formate and methanol concentrations and carbon isotope compositions

The CO_2_ concentration in sub-sampled fluids was consistently lower than the total amount of CO_2_ that was added to the system. This decrease in CO_2_ can be explained by adsorption on the coal, mineral trapping, or consumption by bacteria. Previous reports have suggested that several gases are adsorbed by immature coal (White et al., 2005; Bustin and Clarkson, 1998; Pan and Connell, 2007). The FE-SEM-EDS results of this study suggested that mineral trapping of CO_2_ by carbonate precipitation could have accounted for the slightly lower than expected CO_2_ concentrations. On the other hand, the increase of acetate concentration in the sub-sampled fluids was observed during the experiment, which, in conjunction with decreases in H_2_ and CO_2_ concentrations, implies that homo-acetogenesis occurred during the experiment. The lower δ^13^C value of increased acetate during the experiment than that in normal total organic carbon (−20 to −30‰) also suggests that acetate was produced by homo-acetogenesis. This is because, acetate synthesized via the acetyl-CoA pathway during homo-acetogenesis by acetogen is depleted in ^13^C compared with its precursor (House et al., 2003). Indeed, the presence of *Sporomusa*-related 16S rRNA genes, a homo-acetogenic bacterium, detected in the cloning analysis of post-sand also supports this interpretation. δ^13^C_CO2_ simply reflects the value of supplied CO_2_ and bicarbonate in pore water, which ranged from −24.4 to −19.2‰. A decrease of formate concentrations and enrichment of ^13^C in δ^13^C_formate_ also likely indicate bacterial consumption of formate in which ^12^C would be preferentially consumed.

Interestingly, we observed increase of methanol concentration during the CO_2_ injection experiment (Figure 9; Table 5). The carbon isotopic compositions of methanol were relatively constant at around −40‰ although the δ^13^C values were notably lower than those of DIC. Given the available data set, it is still difficult to identify the source and/or production mechanisms of methanol at this point; however, we infer that continuous injection of the CO_2_ and fluid might abiotically release absorbed methanol from the coal-formation sample. It might also be conceivable that microbial activity stimulated by the CO_2_ and fluid supply might contribute to methanol production (e.g., as a secondary product via degradation of organic matter).

### Homo-acetogenesis vs. methanogenesis

**Figure 10:**
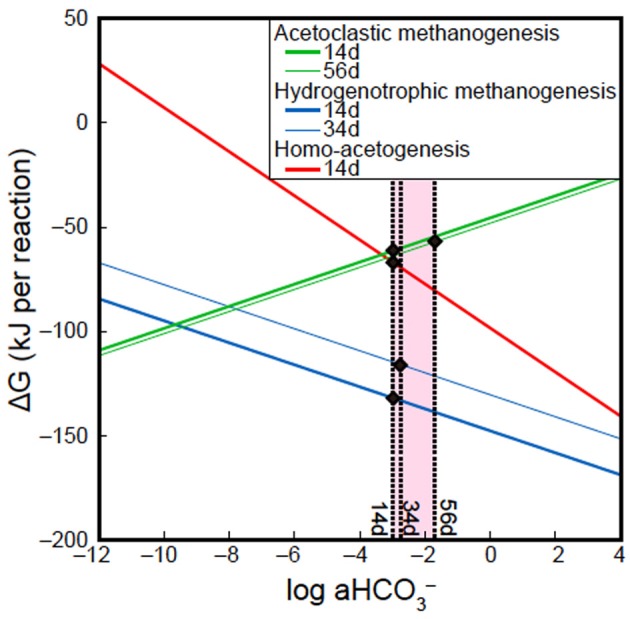
**Effects of HCO^−^_3_ activity (a HCO^−^_3_) on ΔG of acetoclastic methanogenesis, hydrogenotrophic methanogenesis and homo-acetogenesis**. All lines in figure were calculated using geochemical data of samples 14d, 34d, and 56d, except Dissolved inorganic carbon (DIC) concentrations. Solid circles indicate ΔG of acetoclastic methanogenesis, hydrogenotrophic methanogenesis and homo-acetogenesis at the DIC concentrations examined in this study.

Batch-type cultivation of methanogens at ambient temperature and pressure followed by cloning analysis revealed that *Methanobacterium* and *Methanosarcina* were indigenous to the Kushiro bituminous coal examined in this study. This finding is consistent with previous reports describing the presence of methanogens in coal (Krüger et al., 2008; Strapoć et al., 2008). However, no methanogens were activated during CO_2_ injection experiment under *in-situ* subsurface conditions. To constrain the habitability of bacteria and methanogens, we calculated ΔG of acetoclastic methanogenesis, hydrogenotrophic methanogenesis and homo-acetogenesis using *in-situ* geochemical data obtained in the present study (Tables 5, 6; Figure 10). We could not calculate ΔG of acetoclastic methanogenesis on day 34, hydrogenotrophic methanogenesis on day 56, and homo-acetogenesis on days 34 and 56 because the concentrations of acetate in sample 34d and the dissolved H_2_ in sample 56d were below detection limit. On days 14 and 34, the ΔG of hydrogenotrophic methanogenesis was the lowest of all reactions, indicating that hydrogenotrophic methanogenesis is most favorable under the conditions examined (Figure 10). It is thus enigmatic that hydrogenotrophic methanogenesis did not occur, even though methanogens are indigenous to the examined coal and sufficient H_2_ and CO_2_ were supplemented into the coal-sand column during experiment. Slow metabolic rates of methanogens might be responsible for the result. Under conditions of supplemented H_2_ and CO_2_, homo-acetogens and hydrogenotrophic methanogens would compete against each other because both utilize H_2_ and CO_2_. *Sporomusa*, the homo-acetogenic bacterium observed in post-sand, has been reported to be capable of demethylating aromatic compounds and of breaking the bonds of aromatic rings (Mechichi et al., 1999), many of the aromatic organic compounds supplied by pyrolysed/pressurized bituminous coal could be used by *Sporomusa* as nutrients, resulting in the predominance of homo-acetogenesis in the system. Alternatively, despite the sub-sampling and reactor experiment were carried out under the anaerobic condition, we cannot deny the possibility that small oxygen contamination during the initial sampling might negatively affect methanogenesis and if so, it would be outcompeted by homo-acetogenesis under trace oxygen conditions (Leadbetter and Breznak, 1996).

**Table 6:** **Gibbs free energies and enthalpies of possible microbial reactions in CO_2_ injection experiment**.

**Reaction**		**ΔH◦(KJ)**	**ΔG◦(KJ)**	ΔG◦_40, 41_(KJ)^*^	ΔG◦_40, 41_(KJ)^*^		
					**14d**	**34d**	**56d**
Acetoclastic methanogenesis	CH_3_COO + H_2_ O→CH_4_ + HCO^−^_3_	7.1	−31.2	−34.4	−61.2	NA	−56.6
Hydrogenotrophic methanogenesis	HCO^−^_3_ + 4H_2_ + H^+^→CH_4_ + 2H_2_O	−225.7	−246.2	−250.2	−131.8	−114.8	NA
Homo-acetogenesis	2HCO^−^_3_ + 4H_2_ + H^+^→CH_3_COO^−^ + 4H_2_O	−232.8	−215.1	−215.8	−65.4	NA	NA

*ΔH◦ and ΔG◦ represent enthalpy and standard Gibbs free energy (25◦C, 1 atm)*.

* ΔG◦_40, 41_ and

* *ΔG_40, 41_ represents Gibbs free energies at equilibrium and calculated using in-situ geochemical data*.

A previous study suggested that an increase in the partial pressure of CO_2_ could promote acetoclastic methanogenesis in crude oil reservoirs (Mayumi et al., 2013). However, in this study, an increase in DIC concentration and a decrease in the acetate concentration associated with acetoclastic methanogenesis were not observed, indicating that acetoclastic methanogenesis did not occur even though the fluid supplemented into coal-sand column contained sufficient acetate for growth. Higher ΔG values of the acetoclastic methanogenesis on days 14and 56 than those obtained for hydrogenotrophic methanogenesis and homo-acetogenesis is also consistent with our results, which can be explained by differences H_2_ utilization in each system. The influence of dissolved H_2_ concentration on the ΔG of hydrogenotrophic methanogenesis was also observed in this study (blue lines in 14d and 34d; Figure 10). The steep gradient of the curves in Figure 10 suggest that an increase in HCO^−^_3_ activity is more effective for promoting homo-acetogenesis than hydrogenotrophic methanogenesis.

In summary, our results suggest that homo-acetogenesis is possible reaction in GCS settings involving unmineable subsurface coal-sand formations. Aromatic organic compounds supplied by bituminous coal can activate homo-acetogenic bacteria. These findings indicate that microbial conversion of CO_2_ to acetate under subsurface conditions is feasible. However, to gain our knowledge of the potential response of subsurface microbial ecosystem to CO_2_ sequestration, more detailed comparative geochemical and microbiological studies using tracer incubation experiments and high-throughput sequencing will be necessary for *in-situ* and *ex-situ* conditions. In addition, to accelerate the biological CO_2_ conversion to reduced compounds (i.e., Bio-CCS), supply of electron and/or molecular hydrogen would be essential. In this regard, we need to investigate the place where natural H_2_ concentration is remarkably high due to the thermal degradation of organic matter (Head et al., 2003) or other H_2_-producing geologic systems (e.g., serpentinization), or to consider the utilization of natural electric resources for electromethanogenesis (Cheng et al., 2009; Kuramochi et al., 2013). These are our on-going foci.

### Conflict of interest statement

The authors declare that the research was conducted in the absence of any commercial or financial relationships that could be construed as a potential conflict of interest.
